# A Case Report of BRASH (Bradycardia, Renal Failure, Atrioventricular (AV) Blockage, Shock, and Hyperkalemia) Syndrome With a Challenging Diagnosis and Management Dilemma

**DOI:** 10.7759/cureus.46413

**Published:** 2023-10-03

**Authors:** Muhammad Ghallab, Nicole C Noff, Jasmine Sandhu, Alli El-ijla, Karim Makhoul, Asad Sahibzada, Most Munira

**Affiliations:** 1 Internal Medicine, Icahn School of Medicine at Mount Sinai/NYC Health+Hospitals, Queens, New York, USA; 2 Pulmonary and Critical Care, Icahn School of Medicine at Mount Sinai/NYC Health+Hospitals, Queens, New York, USA; 3 Cardiology/Medicine, Icahn School of Medicine at Mount Sinai/NYC Health+Hospitals, Queens, New York, USA

**Keywords:** beta blocker side effects, severe bradycardia, acute kidney injury, severe hyperkalemia, brash syndrome

## Abstract

BRASH syndrome, characterized by bradycardia, renal failure, atrioventricular (AV) blockage, shock, and hyperkalemia, is an emerging clinical entity that challenges healthcare practitioners. This case report presents a unique instance of BRASH syndrome with an atypical presentation in a 56-year-old woman with a past medical history of hypertension, diabetes, and chronic kidney disease. Initial laboratory results revealed severe normocytic anemia, thrombocytopenia, renal dysfunction, acidosis, and hyponatremia, alongside hyperkalemia and hypothyroidism. An electrocardiogram depicted sinus arrest with atrial escape rhythms, indicative of severe bradycardia. Imaging studies revealed pleural effusion and ground glass opacities. Management involved anti-hyperkalemic measures, discontinuation of AV nodal-blocking agents, thyroid hormone replacement, and vasopressor support. The patient eventually improved following continuous renal replacement therapy (CRRT) and hemodialysis. The diagnosis of BRASH syndrome emerged as the most likely due to recurrent admissions with similar clinical features. BRASH syndrome represents a complex interplay between AV nodal block and hyperkalemia, leading to severe bradycardia and shock, often affecting older patients with limited renal reserve. While the current literature primarily consists of case reports, raising awareness of BRASH syndrome is crucial for timely intervention and improved patient outcomes. Further research is needed to better understand the mechanisms underlying this syndrome.

## Introduction

BRASH syndrome, a recently identified clinical entity, is characterized by a pentad of clinical manifestations encompassing bradycardia, renal failure, atrioventricular (AV) blockage, shock, and hyperkalemia [1].

The emergence of BRASH syndrome represents a contemporary addition to the spectrum of clinical syndromes, delineating a multifaceted interplay of symptoms comprising bradycardia, renal failure, the administration of AV node-blocking medications, shock, and hyperkalemia. This novel syndrome predominantly serves to explicate the intricacies surrounding the management of severe bradycardia and shock in patients concurrently afflicted by hyperkalemia while undergoing AV-node blocker therapy. The pathophysiological foundations of BRASH syndrome find their origin in a series of events catalyzed by factors such as hypovolemia, which accentuate renal dysfunction and contribute to the accumulation of AV-node blocking agents and potassium within the system. The identification of BRASH syndrome-associated bradycardia carries profound clinical ramifications, as it may manifest with a more pronounced severity than initially foreseen and may demonstrate resilience against conventional bradycardia treatment protocols, thereby precipitating hemodynamic instability and unfavorable patient prognoses [2].

A significant number of healthcare professionals have effectively managed patients with BRASH syndrome, often without explicitly discerning the specific underlying mechanism. The majority of individuals afflicted by BRASH syndrome typically exhibit positive responses to fundamental supportive interventions [3]. Here, we present a case of BRASH syndrome with an atypical presentation. 

## Case presentation

History of presentation

A 56-year-old woman presented with altered mental status and dizziness. Physical exam was significant for a blood pressure of 90/50 mmHg, heart rate of 20 beats per minute (bpm), sluggish, cold to touch, generalized anasarca, and scattered bilateral rhonchi on auscultation.

Past medical history

A month before this hospitalization, the patient had another admission for acute kidney injury (AKI) and altered mental status, bradycardia, and hyperkalemia. She had her beta-blocker (carvedilol 6.25 mg twice daily) tapered to improve bradycardia while titrating a calcium channel blocker (Amlodipine 10 mg daily) to be used along with hydrochlorothiazide 12.5 mg daily and clonidine 0.1 mg twice daily for blood pressure control. There were also concerns of myxedema coma after her thyroid stimulating hormone levels were significantly elevated (40 IU/mL), and she was placed on oral levothyroxine 112 microg. The patient had comorbidities of type 2 diabetes for 10 years, hypertension for 15 years, hyperlipidemia for 10 years, and chronic kidney disease stage IIIa for three years.

Differential diagnosis

Based on the clinical scenario, the differential diagnoses were symptomatic bradycardia due to myxedema coma, hyperkalemia, or medication toxicity (beta-blocker and clonidine toxicity). In addition, the altered mental status with hypotension was suspected to be due to underlying sepsis or adrenal insufficiency. The hypotension with generalized anasarca raised the suspicion of cardiogenic shock. Based on the recent recurrent admissions with AKI, bradycardia, hyperkalemia, and the recent use of beta blockers, the diagnosis of BRASH syndrome was suspected.

Investigations

Initial blood work (Table 1) was significant for severe normocytic anemia, thrombocytopenia, abnormal kidney functions, non-anion gap metabolic acidosis, hyponatremia, hyperkalemia, and hypothyroidism. Blood sugar was found to be borderline in the lower range (70-90 mg/dL). 

**Table 1:** Summary of the initial blood work done in the emergency department. NT-proBNP, N-terminal pro-brain natriuretic peptide; AST, aspartate aminotransferase; ALT, alanine aminotransferase

Labs	Value	Reference range
Complete blood count		
Hemoglobin (Hb)	7.7 g/dl	12.0-16.0 g/dL
Mean corpuscular volume	84 fL	78.0-95.0 fL
White blood count (WBC)	5.62 x 10(3)/mcL	4.8x 10^3^ to10.8 x 10^3 ^mcL^-1^
Platelets	72 x 10^3 ^mcL^-1^	150 x 10^3^to 450 x 10^3 ^mcL^-1^
Kidney functions tests		
Blood urea nitrogen	80 mg/dL	6-23 mg/dL
Creatinine	1.73 mg/dL	0.5-1.2 mg/dL
Sodium	123 mmol/L	136-145 mmol/L
Potassium	6.4 mmol/L	3.5-5.1 mmol/L
CO_2_	16 mmol/L	26-29 mmol/L
Anion gap	13 meq/L	8-16 meq/L
Liver function tests		
ALT	66 U/L	0-33 U/L
AST	26 U/L	5-32 U/L
Albumin	3.2 g/dL	3.5-5.2 g/dL
Coagulation profile		
D-dimer	7,853 ng/mL	≤285 ng/mL
Activated partial thromboplastin time (aPTT)	48.5 seconds	25.1-36.5 seconds
Prothrombin time (PT)	14.6 seconds	10.0 - 13.0 seconds
Venous blood gas (VBG)		
PH	7.21	7.35-7.45
PCO_2_	40 mmHg	32-35 mmHg
Lactate	5.1 mmol/L	0.4-0.8 mmol/L
Troponin-I	0.016 ng/mL	≤0.010 ng.mL
NT-proBNP	1,172 pg/mL	1-125 pg/mL
Thyroid-stimulating hormone (TSH)	11.5 microIU/mL	0.27-4.20 microIU/mL

**Figure 1:**
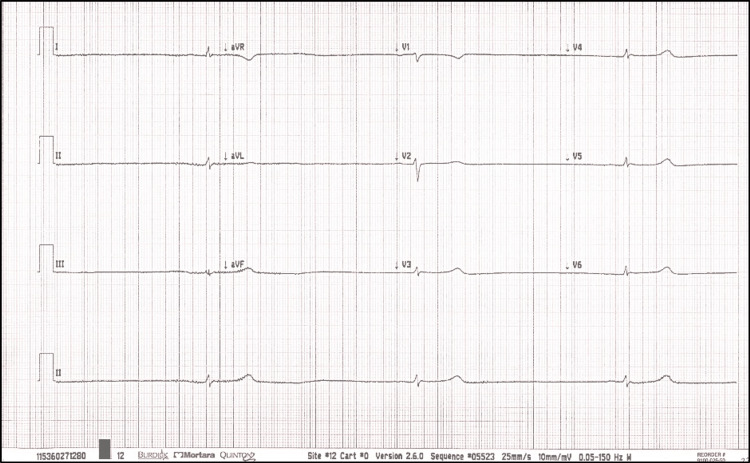
Twelve-lead EKG with a rhythm strip shows sinus arrest with atrial escape rhythm at a rate of 20 beats per minute. EKG, electrocardiogram

An electrocardiogram (EKG) showed (Figure 1) sinus arrest with atrial escape rhythms of 20 bpm.

**Figure 2:**
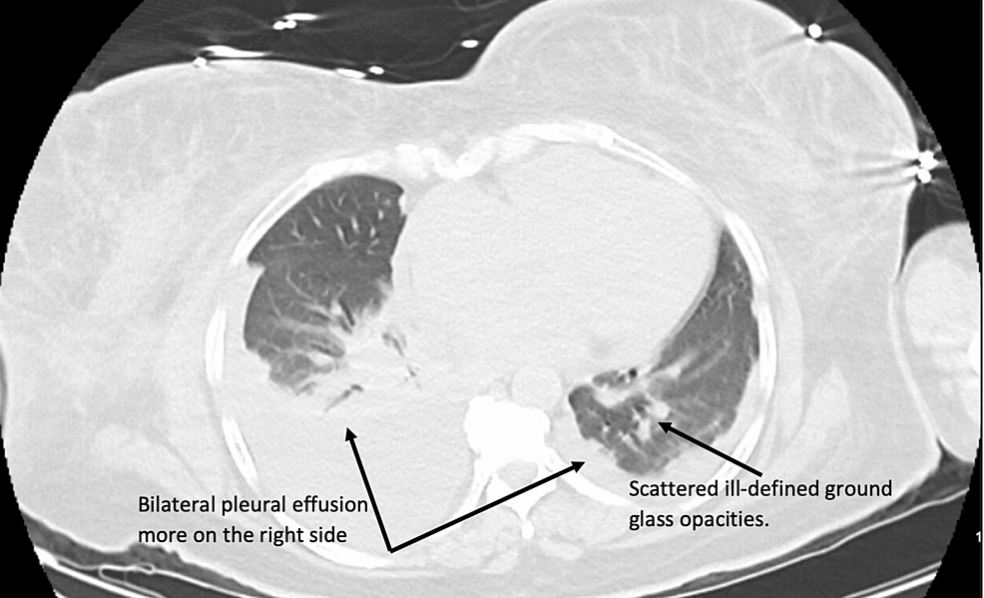
CT chest without contrast shows bilateral pleural effusion more at the right side and scattered ill-defined ground glass opacities suggestive of pneumonia. CT, computed tomography

Trans-thoracic echocardiogram (TTE) was significant for normal cardiac contractility with an ejection fraction of 60%, dilated left and right atria, and small pericardial effusion. Computed tomography (CT) head revealed no acute hemorrhage or infarction. CT chest (Figure 2) without contrast revealed bilateral moderate pleural effusion, more at the right side, scattered ill-defined ground glass opacities suggestive of pneumonia. 

Management

**Figure 3:**
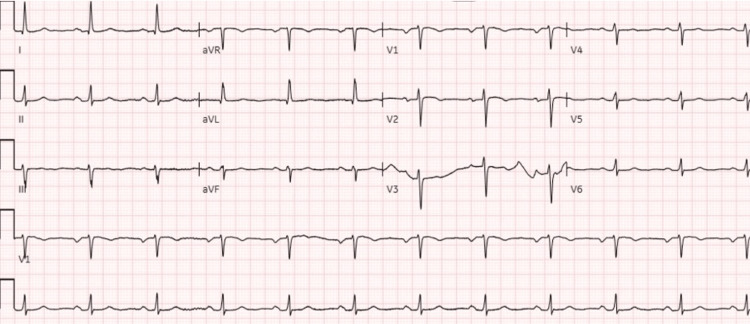
Twelve-lead EKG with a rhythm strip shows normal sinus rhythm at a rate of 68 beats per minute. EKG, electrocardiogram

The patient was given anti-hyperkalemic medications in the form of intravenous (IV) regular insulin and dextrose 25%, inhaled beta-2 agonist, and calcium gluconate. The potassium level improved to 4.1 mmol/L, yet the patient remained bradycardic. The patient was placed on epinephrine and glucagon drip with the discontinuation of the beta blocker and clonidine. IV levothyroxine was initiated for possible myxedema coma, and IV hydrocortisone was given for possible adrenal insufficiency, but the diagnosis was excluded after the cortisol level was found to be normal. A bicarbonate drip was started for metabolic acidosis, and a furosemide infusion was started for volume overload. The heart rate improved to 50-60 bpm, repeat EKG showed normal sinus rhythm (Figure 3), and blood pressure improved to 110/60 mmHg.

The patient was placed on broad-spectrum IV antibiotics for presumed sepsis. Although the patient was being maintained on appropriate management, her mental status worsened, requiring intubation for airway protection. The patient later developed oliguria and failed a diuretic challenge; therefore, continuous renal replacement therapy (CRRT) was initiated. After two days on CRRT, the patient's hemodynamics improved, the vasopressors were tapered, and then placed on intermittent hemodialysis for two days and extubated successfully. Given the patient's history of recurrent admissions with hyperkalemia, renal failure, and shock with bradycardia in the AV nodal blocking agents setting, with improvement on hemodialysis and discontinuation of the AV nodal blocking agents, diagnosis with the BRASH syndrome was the most likely diagnosis.

During the rest of the hospital stay, the patient was placed on IV furosemide and metolazone and maintained adequate urine output with the improvement of kidney functions and discontinuation of the hemodialysis. The patient was then discharged in stable condition on hydralazine for blood pressure control with the discontinuation of the beta-blockers and clonidine.

## Discussion

This case report describes a rare case of BRASH syndrome characterized by bradycardia, renal failure, AV nodal blocking agents, shock, and hyperkalemia. 

The pathophysiology of BRASH syndrome remains unclear; however, it is likely a synergistic effect between AV nodal block and hyperkalemia, resulting in severe bradycardia. Patients on AV nodal-blocking agents with chronic kidney disease can develop worsening renal function with hyperkalemia, leading to bradycardia. This progression will reduce cardiac output, prompting renal hypoperfusion and dysfunction with consequent shock. The kidney injury will then further propagate hyperkalemia. This vicious pathophysiological cycle is now classified as BRASH syndrome [1]. This condition is most common in older patients with cardiac disease and limited renal reserve, as it involves antihypertensive medications and reduced renal function [4]. The proposed underlying pathophysiology includes a triggering event such as hypovolemia that will worsen renal function and result in the accumulation of AV nodal-blocking agents and potassium [2]. Other triggering events include medications such as Ranolazine and Bactrim, anaphylaxis, and COVID-19 [3].

The BRASH syndrome is the middle ground between isolated hyperkalemia and isolated AV nodal-blocking agent overdose, and it is not always differentiable. Isolated hyperkalemia generally will not precipitate bradycardia unless severe and >7 meq/L. An EKG revealing bradycardia without characteristic findings of hyperkalemia will suggest an additional contributing factor. Thus, moderate hyperkalemia with bradycardia can be differentiated as BRASH syndrome based on the presence of AV nodal-blocking agents. As such, clinical history is imperative to the diagnosis [4]. 

Patients with the BRASH syndrome frequently comply with correct medication dosing and rarely have supra-therapeutic blood levels of AV nodal-blocking agents [1]. The problem arises due to the synergy between drug levels and hyperkalemia. Another feature favoring BRASH syndrome is hyperkalemia's dramatic clinical response to intravenous calcium administration [4]. 

The current data on BRASH syndrome consists mainly of case reports, which are still broadly unknown and underdiagnosed. A retrospective analysis from the emergency department of the Buergerspital Solothurn between January 1, 2017, and December 31, 2018, of age 18 years or above revealed a total of only 8 out of 65,489 patients reviewed (prevalence of 0.04%) who presented with hyperkalemia, AKI, bradycardia, and systolic hypotension and were taking AV-node blocking agents and thus fulfilled the BRASH criteria [2]. 

The goal of treatment is to correct hyperkalemia, provide hemodynamic support for bradycardia and hypotension, and treat any triggering event, such as hypovolemia or atrioventricular nodal-blocking agents [1]. If hyperkalemia is severe, aggressive diuresis may be attempted. Epinephrine will increase heart rate and cardiac output, improving hemodynamics and renal perfusion while simultaneously shifting potassium intracellularly. Advanced therapies to consider are beta-blockers and calcium channel blocker reversal agents. The foremost important part of treatment is not any single intervention; rather, concurrently addressing several problems [4]. The most common error in treatment is overlooking other aspects of the syndrome while focusing on a single component, such as hyperkalemia. The syndrome can be severe and refractory to standard bradycardia therapies, so early diagnosis is crucial to prevent the progression of hemodynamic instability and mortality [2]. If medical treatments fail, transvenous pacing and hemodialysis may be necessary [4]. In many cases, the BRASH syndrome can often go undiagnosed and still be successfully managed with supportive care [3]. 

## Conclusions

It's crucial to consider BRASH syndrome in the differential diagnosis of patients presenting with bradycardia, AKI, hyperkalemia, and recent AV nodal-blocking agents intake. This consideration is essential to direct the management properly, especially if the presentation is life-threatening, as in our patient with severe bradycardia and shock. More studies in the future are needed to understand the mechanism of this syndrome better. 
